# Helping feed the world with rice innovations: CGIAR research adoption and socioeconomic impact on farmers

**DOI:** 10.1016/j.gfs.2022.100628

**Published:** 2022-06

**Authors:** Ashok K. Mishra, Valerien O. Pede, Aminou Arouna, Ricardo Labarta, Robert Andrade, Prakash C. Veettil, Humnath Bhandari, Alice G. Laborte, Jean Balie, Bas Bouman

**Affiliations:** a Kemper and Ethel Marley Foundation Chair, Morrison School of Agribusiness, W. P. Carey School of Business, Arizona State University, Mesa, AZ, 85212, USA; b International Rice Research Institute, Los Banõs, Philippines; c Africa Rice Centre (AfricaRice), Bouake, Republic of Côte d'Ivoire; d Formerly International Center for Tropical Agriculture (CIAT), Cali, Colombia; e Alliance of Bioversity International and CIAT, Cali, Colombia

**Keywords:** Impact assessment, Natural resource management, Latin America, Southeast and South Asia, Africa, Post-harvest and other technologies, Direct-seeded rice

## Abstract

Rice production has increased significantly with the efforts of international research centers and national governments in the past five decades. Nonetheless, productivity improvement still needs to accelerate in the coming years to feed the growing population that depends on rice for calories and nutrients. This challenge is compounded by the increasing scarcity of natural resources such as water and farmland. This article reviews 17 ex-post impact assessment studies published from 2016 to 2021 on rice varieties, agronomic practices, institutional arrangements, information and communication technologies, and post-harvest technologies used by rice farmers. From the review of these selected studies, we found that stress-tolerant varieties in Asia and Africa significantly increased rice yield and income. Additionally, institutional innovations, training, and natural resource management practices, such as direct-seeded rice, rodent control, and iron-toxicity removal, have had a considerable positive effect on smallholder rice farmers’ economic well-being (income and rice yield). Additional positive impacts are expected from the important uptake of stress-tolerant varieties documented in several Asian, Latin American, and African countries.

## Highlights

• The study reviews ex-post impact assessment studies on improved rice varieties.
• Articles were published between 2016 and 2021.
• Stress-tolerant varieties significantly affect rice yields and income of smallholders.
• Institutional innovations and training programs show impact on income and rice yields.
• Direct-seeded rice, rodent control, and iron-toxicity removal significantly affect economic well-being.

## 1 Introduction

Rice, a staple food for some 4 billion people globally, provides 21 percent of global human per capita energy and 15 percent of per capita protein (United Nations, 2017). Rice is grown in diverse agro-ecosystems in South Asia, Southeast Asia, Africa, and Latin America. It is one of the Green Revolution crops that benefited from genetic improvement. The first decade of the 21st century has witnessed slower growth in rice's area, output, and productivity around the globe. Rice farming is associated with poverty in many areas. About 900 million of the world's poor depend on rice as producers or consumers. Finally, about 400 million poor and undernourished people are engaged in rice-based farming systems, mostly on less than 20 ha of landholding. A report by the CGIAR^1^ System notes that with expected growth in population and income and a decline in rice acreage, global demand for rice will continue to increase from 479 million tons of milled rice in 2014 to between 536 million and 551 million tons in 2030, with little scope for easy expansion of agricultural land or irrigation (except for some areas in Africa and South America).

About 144 million farm households, usually small farms, are engaged in rice production for subsistence and employment. To meet the challenges of production and employment, the CGIAR group has invested heavily in researching and developing new technologies (new varieties created through genetic improvement, natural resource management, and post-harvest technologies) for the past seven decades. Rice innovations have decreased food shortages and increased rice yields in many countries. Most studies that demonstrated the reach and effects of CGIAR rice-related innovations were published by 2015 (Yamano et al., 2016). However, Yamano et al.’s (2016) study reviewed research results focusing on crop genetic improvement and crop management resources. Rice technologies are continuously changing, and earlier studies did not capture newer and advanced technologies developed after 2016.

Thus, this review article contributes to documenting the reach and impacts of CGIAR-related rice technologies, whose contributions have not been updated since 2015. This review focuses mainly on rice innovations developed or scaled by CGIAR centers and partners during the implementation of the CGIAR research programs (Global Rice Science Partnership [GriSP] and Rice Agri-Food System Researh Program or RICE) in rice and on an impact assessment of peer-reviewed studies completed from 2016 to 2021. We used the following criteria for selecting studies in this article. First, we included (1) rice technologies developed in CGIAR, particularly at the International Rice Research Institute (IRRI), the International Center for Tropical Agriculture (CIAT), and AfricaRice, in collaboration with their partners; (2) technologies that are mature and already being used by rice value chain actors; (3) studies using quantitative or qualitative methods of evaluation in their assessment^2^; and (4) only ex-post adoption and impact assessment studies. Second, we excluded: (1) technologies in the process of research and development or still being tested; (2) technologies developed by the private sector; (3) technologies developed or promoted outside of the international research centers listed above; and (4) the impact of climate change and COVID-19 shocks. In reviewing a broad range of adoption and impact valuation studies in rice innovations developed by the CGIAR system, we hope to provide a roadmap for future studies. This article is not a comprehensive impact assessment of the whole range of rice research and development, novel technologies, and management practices of the CGIAR rice research. Instead, the study is a selection of impact studies conducted by CGIAR centers.

The rest of this paper is organized as follows. Section 2 presents rice research in the CGIAR system. Section 3 reviews articles on the adoption and impacts of improved rice varieties. Section 4 offers the reviews of articles on the adoption and impact of improved natural resource management practices (such as crop establishment methods, post-harvest technologies, information and communication technologies, and institution and training programs). Section 5 provides the limitations of this study and discusses knowledge gaps. The last section provides conclusions.

## 2 Rice research in CGIAR

**Fig. 1:**
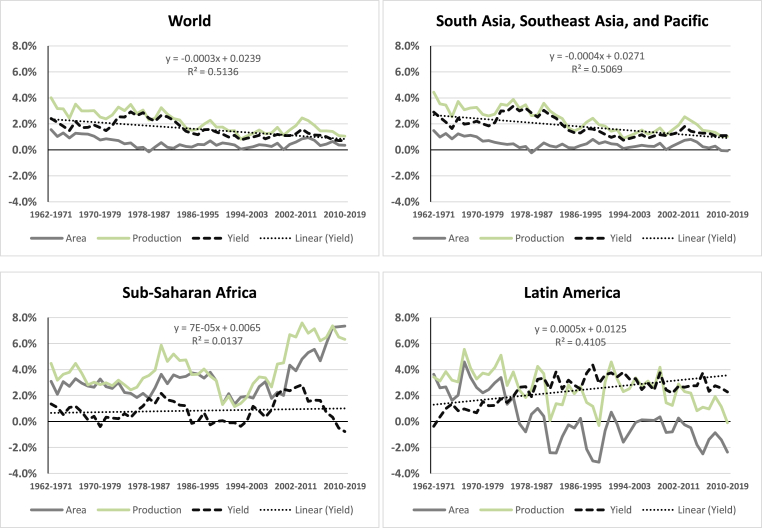
Average growth rate (%) of area (hectares), production (tons), and rice yield (t/ha), by period, 1962–2019.

Rice is one of the crops that played a critical role in rapidly reducing food insecurity in the 1960s through the Green Revolution. Fig. 1 shows the growth rate of rice area, production, and yield from 1962 to 2019 for the world and three rice-growing regions (Latin America, South and Southeast Asia and the Pacific, and sub-Saharan Africa). The figure shows that high growth was apparent during the Green Revolution (1966–1985), with world rice production and yield increasing by 3% per year. However, the rice area increased by less than 1% in each period and has decreased in recent years. We observe a similar trend in South and Southeast Asia. In fact, over the past 20 years, the total area of land used for rice farming has been declining (Fig. 1, top-right panel). Similarly, we see a negative growth rate in rice in Latin American countries (Fig. 1, bottom-right panel), where the decrease was more pronounced than in any other region. Remarkably, the average growth rate in rice yield in Latin America (about 2.5% in 2008–2019) was the highest of all the regions. In contrast, Fig. 1 (bottom-left panel) shows that growth in rice areas was highest in sub-Saharan Africa (7.3% during 2008–2019) relative to other regions. However, the growth in rice yield in sub-Saharan Africa was reversed and turned negative in the later period (2008–2019), from 0.3% to −0.8%, relative to other regions.

Table 1 shows that GDP per capita income in Asia increased from 1970 to 2018. The share of rice as the primary source of income in Asia decreased from 26% in 1970 to 13% in 2018. This change can be attributed to labor moving to non-farm sectors (Pandey et al., 2010; Emerick, 2018). Emerick (2018) found that a short-term increase in agricultural productivity in India led to decreases in farm labor and increases in the amount of labor in the non-farming sector. In addition, government programs have facilitated the movement of family labor to the non-rural farm sectors. Meanwhile, in Latin America and sub-Sarahan Africa, rice consumption has increased rapidly as GDP per capita increases in (Table 1). A plausible explanation could be that, as observed in Latin America, sub-Saharan Africa (Nigatu et al., 2017) and Asian (Mottaleb et al., 2017) consumers demand better-quality rice and, in some cases, more nutritious rice (such as biofortified rice), which fetches higher market prices for farmers and increases spending by consumers. In addition, changes in consumption patterns occurred in the Asian region. Table 2 shows mixed patterns in rice consumption between rice-producing regions. Timmer et al. (2010) found several rice consumption patterns in Asia. These patterns can be attributed to several factors, such as a diversity of rice consumption within a country and across countries, differences in consumption by income class, differences in consumption in rural and urban areas, and changing tastes and preferences among consumers in urban areas. Increases in rice yield and income have been closely linked to better-quality rice consumption (Yamano et al., 2016). Thus, rice can play an essential role in feeding the world, and rice research can inform the best strategies to keep rice at the heart of global food security.

**Table 1:** Importance of rice as a source of food and income by region.

Type	GDP per capita income USD (value of USD per capita)	Rice for domestic consumption (million tons)^a^	Food supply (kg/capita/yr)^c^	Rice production milled (million tons)^a^	Percentage of value of rice in the total value of agriculture^b^
Africa					
1970	320.87	5.11	10.85	4.83	2.86
1980	1283.62	7.53	14.88	5.27	2.91
1990	937.62	11.15	16.75	7.76	3.09
2000	1912.15	16.08	18.76	11.15	4.60
2018	1858.78	37.64	34.26	33.48	3.27
South America					
1970	606.89	6.07	26.98	5.43	3.09
1980	1977.40	9.28	29.47	8.96	3.36
1990	2588.92	10.87	31.58	10.13	2.57
2000	9804.27	12.89	30.43	12.84	3.09
2018	6944.07	14.89	44.74	23.74	2.07
Asia					
1970	245.51	191.70	77.45	195.90	26.63
1980	967.01	241.90	77.20	244.94	24.34
1990	1699.57	308.36	82.40	321.71	22.24
2000	4901.91	346.68	78.62	362.53	18.48
2018	8786.51	407.41	112.99	662.93	12.83
World					
1970	922.94	209.88	47.61	213.01	11.99
1980	2759.64	270.19	49.71	269.91	11.47
1990	4316.57	343.81	54.22	351.37	11.89
2000	9548.35	393.68	53.69	399.19	10.64
2018	11,244.09	444.12	78.46	742.05	7.93

a Asia consists of South Asia, Southeast Asia, and East Asia, while Africa is composed of countries in sub-Saharan Africa and North Africa.

b Gross value of production (constant 2014–2016 thousand USD).

c For 2018, estimates include rice and products as compared to earlier estimates of milled rice.

**Table 2:** Average growth rates (%) of production (1000 Mt) and consumption (1000 Mt) of milled rice, 1968–2019.

Period	South America		South Asia		Southeast Asia		Sub-Saharan Africa		World	
	Prod	Cons	Prod	Cons	Prod	Cons	Prod	Cons	Prod	Cons
1968–1980	3.98	4.61	3.13	1.44	4.07	2.91	2.07	5.15	2.86	2.71
1981–1990	1.80	2.10	3.35	4.01	2.66	3.07	5.11	4.18	2.75	2.73
1991–2000	2.85	1.70	1.94	1.75	3.00	2.71	2.55	3.12	1.36	1.63
2001–2010	1.34	1.35	1.17	1.42	1.76	1.25	4.17	5.42	0.81	1.02
2011–2019	−0.54	0.26	2.32	1.32	0.23	0.56	4.92	5.69	1.10	1.10
All years	2.39	1.82	2.54	2.12	2.45	1.97	4.07	4.96	1.93	1.75

The three institutions mainly involved in rice research under CGIAR are the International Rice Research Institute (IRRI), AfricaRice, and the International Center for Tropical Agriculture (known by its Spanish acronym, CIAT). These institutions were part of the Global Rice Science Partnership (GRiSP) from 2011 to 2015 to increase rice productivity by using more sustainable rice-based production and by improving efficiency and equity in the rice farming sector. In 2016, the CGIAR Research Program on Rice Agri-Food Systems (the RICE program) was created to follow the Global Rice Science Partnership. However, the goal of each institution remains the same when it comes to developing new rice technologies, but with the added caveat that technologies have to be developed with sustainable natural resource management, including water and land management. Each of the three institutions mentioned above undertakes rice research with a specific regional focus.

The International Rice Research Institute, one of the centers that pioneered rice technologies, was founded in 1960 through the Ford and Rockefeller foundations and is located in the Philippines (Hossain et al., 2003). The institute's primary goal is to “*improve the health and welfare of rice farmers and consumers, promote environmental sustainability in a world challenged by climate change, and* support *the empowerment of women and youth in the rice industry”* (https://www.irri.org) (CGIAR^1998^). Its rice research is also responsible for developing rice varieties, including semi-dwarf and high-yielding IR8, and promoting the use of good agronomic practices (Mackill and Khush, 2018), which paved the way for the Green Revolution in Asia. By 2010, an estimated 480 rice varieties directly linked to the institute had been released in the Philippines, Indonesia, and Vietnam. Brennan and Malabayabas (2011) evaluated the impact of rice varietal improvement on production in three Southeast Asian countries (the Philippines, Indonesia, and Vietnam) from 1985 to 2009, which is estimated to have a net present value (at 2019 USD) of USD 4.2 billion to USD 6.8 billion.

Africa Rice Center, with headquarters in Côte d’Ivoire, was established in 1971, and its membership now comprises 28 African countries. The center's mission is “*to contribute to poverty alleviation and food security in Africa through research, development, and partnership activities aimed at increasing the productivity and profitability of the rice sector in ways that ensure the sustainability of the farming environment*” (Africarice.org, 2021). One of AfricaRice's significant achievements was the development of New Rice for Africa (NERICA) in 1994. NERICA varieties were created by a cross between Asian rice (*Oryza sativa*) and African rice (*Oryza glaberrima*). A total of 328 varieties, including 82 NERICA varieties, were released, mainly composed of varieties for rain-fed lowlands with a yield potential of 6–7 tons per hectare (Africarice.org, 2021).

Another CGIAR member organization involved in rice research is the International Center for Tropical Agriculture, the third CGIAR member engaged in rice research, established in 1967 and located in Colombia. The center's mission is to “*increase prosperity and improve human nutrition in the tropics through research-based solutions in agriculture and the environment*” (CIAT, 2021). Besides beans, cassava, and tropical forages, the center focuses on rice, collaborating with the Latin American Fund for Irrigated Rice (Hossain et al., 2003). By 2017, the center had released 377 rice varieties with the center's DNA in their pedigree in Latin America and the Caribbean (CIAT, 2017).

In sum, CGIAR research developed or scaled up during the GRiSP/RICE era was instrumental in informing the scaling strategies adopted by CGIAR and its partners. The CGIAR centers involved in rice research worked together to maximize the system's contributions toward a better-nourished world.

## 3 Improved rice varieties: adoption and impact

Farmers and smallholders worldwide increasingly face declining yields due to climate-related stress and biotic and abiotic stress. CGIAR centers, through innovative and precision breeding tools, have developed rice varieties to address yield reduction and yield stagnation. Smallholder rice farmers have increased their farm incomes by growing novel varieties with higher yield (under stress and climate change), more stable yield, and more efficient use of resources. This section discusses the adoption and impact of rice varieties developed by CGIAR centers to withstand climatological stresses and resistance to pests and diseases in rice production (Table 3).

**Table 3:** List of reviewed studies (2016–2021).

Authors	Year	Countries	Technologies	Improved rice varieties	Natural resource management	Agronomic practices	Post-harvest technologies	ICT and decisions	Training and institution
Nihn et al.	2016	Vietnam	Ecologically based rodent management		✔	✔			
Yorobe et al.	2016	Philippines	Green Super Rice varieties	✔					
Arouna et al.	2017	16 SSA countries	Improved rice varieties	✔					
Dibba et al.	2017	The Gambia	Improved rice varieties	✔					
Arouna	2018	Benin	Collective marketing						✔
Mishra et al.	2018	India	Direct-seeded rice		✔				
Nakano et al.	2018	Tanzania	Farmer-to-farmer extension					✔	✔
Arouna and Akpa	2019	Benin and Togo	Smart-valley approach		✔	✔			
Sha et al.	2019	China	Direct-seeded rice		✔				
Ogwike et al.	2020	Senegal	ASI thresher technology			✔	✔		
Saito et al.	2020	Côte d’Ivoire	Lowland rice variety WITA 9	✔					
Paik et al.	2020	Vietnam	Salt-tolerant rice varities	✔					
Villanueva et al.	2020	India	Varietal improvement by International Rice Genebank	✔					
Arouna et al.	2021	Nigeria	Personalized extension advice					✔	
Arouna et al.	2021	Benin	Contract farming						✔
Veettil et al.	2021	India	Climate-smart varieties	✔					
Bairagi et al.	2021	Bangladesh	Submergence-tolerant rice varieties	✔					
**Total studies**	**17**								

### 3.1 Climatological stress-tolerant rice varieties

Emerick et al. (2016) argue that technological progress in agriculture may be responsible for the lack of adoption of improved farming practices and modern inputs. Although the Green Revolution increased rice production in India, the authors conclude that new research should focus on rice seed varieties that should be well adapted to local conditions, such as drought- and flood-tolerant varieties. Recent literature points to the need for farm field days for increasing the adoption rate for the flood-tolerant rice variety Swarna-Sub1. The notion is that farmers need to be convinced before adopting the technology. In a recent study in India, Emerick and Dar (2021) make a case for farmer field days to increase rice technology adoption. The authors found that farmer field days increased the adoption rate of flood-tolerant Swarna-Sub1 by 40%.

Since 2016, several rice varieties have been developed and disseminated in Asia, Latin America, and Africa. The International Rice Research Institute has developed drought-tolerant varieties that have been released in several countries and now are being planted by farmers, such as the varieties Sahbhagi dhan in India, Sahod ulan in the Philippines, and Sookha dhan in Nepal. Field trials suggest that drought-tolerant varieties have an average yield advantage over drought-susceptible ones of 0.8–1.2 tons per hectare under drought conditions. In the Philippines, field trials of Salinas suggest a yield advantage of at least 2 tons per hectare over non-tolerant varieties. A gene for salinity tolerance, called Saltol, has been incorporated into popular rice varieties in countries across Asia.

In Latin America, newly released varieties are resistant to rice blast and have better processing quality. These varieties, released after 2010, have started to become the dominant cultivars in Colombia (Fedearroz 67 and 68), Peru (La Esperanza), Ecuador (SFL09 and SFL011), Bolivia (MAC 18), and Panama (IDIAP FL 137) (Urioste et al., 2019; Martinez et al., 2021; Marín et al., 2018). In Africa, using drought-tolerant rice varieties is a potential strategy to mitigate the consequences of climate change associated with the increased probability of drought and longer heatwaves. Drought-tolerant rice varieties are expected to produce about 30% of their potential yield after spending six weeks under drought before and during flowering and grain-filling. Several drought-tolerant rice varieties have been developed and introduced in sub-Saharan Africa. ARICA 16 and NERICA 4 were released in Benin in 2013; FARO 58, 59, 63, 64, and 65 were released in Nigeria from 2011 to 2015; and NERICA 4 was released in 2015 in Madagascar.

Similarly, AfricaRice and its national partners developed ARICA 6 to deal with iron toxicity, one of the most important abiotic stresses that limit rice production in sub-Saharan Africa lowlands (AfricaRice, 2014). Iron toxicity increases the sterility rate of panicles. It also decreases yield by 10% to as much as 100%, depending on toxicity intensity, tolerance of the seed, and soil fertility (Audebert and Fofana, 2009; Sahrawat, 2010). This stress affects up to 60% of West and Central Africa (Wopereis et al., 2008; Chérif et al., 2009; Dramé et al., 2010). Rice yellow mottle virus (RYMV) is one of Côte d’Ivoire's main biotic constraints in lowland rice production. The International Institute of Tropical Agriculture (IITA) and the West Africa Rice Development Association (WARDA, renamed AfricaRice) developed a variety, WITA 9 (TOX 3058-28-1-1-1), to mitigate the consequences of rice yellow mottle virus. This variety also increases yield, tolerates iron toxicity and drought, and resists bacterial leaf blight (Singh et al., 2004). Saito et al. (2019) examined the effects of adopting WITA 9 on rice farmers' income and found two main results. First, the authors used a market study to show that consumers were willing to pay more for WITA 9 than all other locally produced rice varieties. The market study also found that the willingness to pay for WITA 9 was similar to that of imported rice. Second, using a survey of 304 households, the authors found that the adoption rate of WITA 9 was about 24%, that the variety increased rice yield by 0.7 tons per hectare, and that the income of farmers who adopted the variety increased by USD 91 per hectare per season.

In another study, Arouna et al. (2017) classified improved rice varieties into two groups: NERICA varieties and all other improved rice varieties (AORV) developed by national or international research institutions and cultivated in sub-Saharan African countries. Arouna et al. (2017) analyzed microdata from 16 of these countries to estimate the effects of adopting improved rice varieties. Using the endogenous switching regression (ESR) framework that controls selection bias, Arouna et al. (2017) found that the adoption rate of enhanced varieties increased from 2000 to 2014, particularly after the 2008 food crisis. Those authors estimated that farmers using NERICA or all other improved rice varieties increased rice yield from 0.15 to 0.75 tons per hectare and increased rice income by USD 20 to 70 per capita. The impact on rice yield and income varied across countries. Similarly, Dibba et al. (2017) investigated the effects of Gambian rice farmers’ adoption of NERICA rice varieties on food security (using food consumption scores).^3^ The authors found that adopting NERICA varieties increased household food security by 14 percentage points.

Flooding is a natural disaster that affects smallholder rice farmers. In India, for example, floods affect 10 million to 12 million of the 20 million hectares of rain-fed rice every year (NRAA, 2013). The International Rice Research Institute discovered *Sub1*, the gene for flood tolerance, and incorporated this gene into popular rice varieties to counteract flood risk (Neeraja et al., 2007; Septiningsih et al., 2009). Submergence-tolerant varieties (Sub1 varieties) can survive at least 7–14 days underwater (Mackill et al., 2012; Rahman and Zhang, 2016). Sub1 varieties have been released and are now being planted across Asia. These include IR64-Sub1 in the Philippines, Swarna-Sub1 in India, Samba Mahsuri-Sub1 in Bangladesh, and Ciherang-Sub1 in Indonesia.

In a recent study, Veettil et al. (2021) analyzed the impact of adopting Swarna-Sub1 (SS1) on rice production and the income of smallholder rice farmers. The authors used a sample of 4744 rice-farming households from three eastern Indian states (Assam, Odisha, and West Bengal). They used the propensity score matching method (PSM) to mitigate biases from observable factors. The authors found that access to information about submergence-tolerant varieties increased the adoption of Swarna-Sub1. With back-of-the-envelope calculations, Veettil et al. (2021) found that the adoption of this variety increased rice production (by 6.25 million tons in all three states) and income (by USD 500 million per year at the state level) in the three eastern Indian states. Other studies found no significant differences in grain yield under stress and quality between Swarna-Sub1 and Swarna, the most popular Indian rice variety (Sarkar et al., 2006; Neeraja et al., 2007).

The most common adverse climatic events in rice farming in Southeast Asia are monsoon and flash floods (Dewan, 2015; Rahman and Zhang, 2016). For example, studies estimate that about 4% of the total rice production in Bangladesh is lost every year because of floods (Paul and Rashid, 1993). The Sub1 rice varieties significantly outperform traditional rice varieties in yield after flooding (Sarkar et al., 2006, 2009; Neeraja et al., 2007; Dar et al., 2013) and have equal yield under no stress. In a recent study, Bairagi et al. (2021) estimated the impact of using Sub1 rice varieties on adopters' farm profit and consumption expenditures in Bangladesh. The authors analyzed a survey of 1625 farmers and used the endogenous switching regression approach to mitigate biases from observable and unobservable factors. They found that rice farmers adopting Sub1 varieties increased farm profit and consumption expenditures but only 40% of northwest Bangladesh's rice farmers adopted Sub1 rice. The authors found that adopters' profit increased by about BDT 20,700 per hectare (or USD 262 per hectare; USD 1 = BDT 79 in 2016). Additionally, per capita consumption expenditures for adopters increased by about BDT 2000 (USD 25).

Yorobe et al. (2016) estimated the impact of Green super rice (GSR) cultivars on yield and income at the farm level in the Philippines. Yorobe et al. (2016) used propensity score matching and difference-in-difference methods and two years of panel data from 138 rice-producing households in the Philippines. The authors found that the adoption of GSR varieties increased yield (by about 1.5 t/ons per hectare) and income (by about PHP 9400 per hectare)^4^ for GSR variety users, particularly in areas with high rainfall. Finally, Paik et al. (2020)Paik et al. (2020) evaluated the adoption and impact of saline-tolerant rice varieties through the Consortium for Unfavorable Rice Environments (CURE)^5^ in Vietnam's Mekong River Delta. The authors found that CURE-related rice varieties are more likely to be adopted in areas with high salinity risk than in areas with low salinity risk. However, the authors found no significant difference in yield under stress and revenues between farmers who grew CURE-related and those who grew other varieties after controlling for farm and household attributes. A plausible explanation could be that the study was completed with no major salinity intrusion event. Therefore, the study could not observe the innovation's full potential.

### 3.2 Pest- and disease-tolerant varieties

The increased volume and intensity of rice production and asynchronous planting of crops have resulted in an increased infestation of rodents. Rodent infestation is one of the top three problems agricultural producers face in Vietnam (Huynh, 1987). It harms rice production in Vietnam significantly, damaging about 10% of the total output every year (Singleton, 2003). Farmers rely on pesticides to control rodent damage, thus exposing non-target species and the environment to risks and decreasing returns on investment (Singleton, 2003). To solve this problem, ecologically based rodent management started in Vietnam in 1996. Ecologically based rodent management aims to increase farmers’ capacity to manage rodent populations and to decrease rice yield losses (Brown et al., 2006).

The approach involves two sets of actions to mitigate rodent pests. The first is community action, consisting of synchronized cropping, field and village hygiene, and rat hunts at critical times. The second component consists of community trap barrier systems (CBTS), which require installing plastic fences set with rat traps around small areas of early-planted rice. Adopting community action is less costly than implementing community trap barrier systems. Nihn et al. (2016) studied the impact of ecologically based rodent management strategies on the yield and income of rice farmers in Vietnam's Mekong River Delta. The authors used a propensity score matching approach to control for biases in observable factors. They found that adopting the community action part of ecologically based rodent management increased rice yield by 0.43–0.45 tons per hectare and real net income by approximately USD 65–67 per hectare for rice farmers in the Mekong River Delta. Nihn et al. (2016) concluded that adopting the community action activities of ecologically based rodent management contributed to food security and environmental improvement in the study area of Vietnam.

In the first decade of the 21st century, Wassmann et al. (2009, 2010) extensively reviewed the consequences of climate change for rice production and possible adaptation strategies of rice farmers. To this end, the Chinese Academy of Agricultural Sciences and the International Rice Research Institute partnered to tackle this problem. This partnership has developed Green Super Rice (GSR) cultivars with high yield, tolerance of salinity or submergence, and resistance to multiple diseases and insect pests. The project's objective was to create rice varieties that maintain stable and sustainable yields under fewer inputs or climatic stresses. The project resulted in new, high-yielding Green Super Rice cultivars tolerant of multiple abiotic stresses (Ali et al., 2013).

In sum, the above studies show that stress-tolerant rice varieties have significantly improved smallholders’ income and rice yields in Asia, Africa, and Latin America. On the other hand, pest- and disease-tolorent rice varieties and their adoption by smallholders show mixed results. Finally, studies in Africa show that consumers are willing to pay more for new rice varieties compared with traditional locally produced rice varieties.

## 4 Improved natural resource management practices: adoption and impact

Improved crop productivity could be obtained by genetic improvement and/or by improved crop and natural resource management. Sustainable natural resource management practices like decreasing water usage through the establishment of direct seeding crop water management, and post-harvest technologies are paramount for rice farmers (Table 3). For instance, using a sample of 8,640 farmers from all over India, Villanueva et al. (2020) examined the link between the genetic contribution of International Rice Genebank (IRG) genes and the yield of improved rice varieties. The authors estimated production functions and found that “a 10% increase in the genetic contribution of IRG accessions to an improved rice variety increased yields by 27%.” Smallholder households' decision-making on technology and natural resource management significantly impacts the cost of production, yield gains, and farming income. Information gathering, training programs, and marketing also affect farmers’ livelihood and incomes. This section discusses the adoption and impact of natural resource management practices developed by CGIAR centers and their effect on rice yield and income of smallholder rice producers.

### 4.1 Crop establishment

Ensuring food security for the growing population in India and most South Asian countries while sustaining agricultural systems under the current scenario of depleting natural resources, increasing input costs, and climate variability calls for a paradigm shift in farming practices. In a study, Suryavanshi et al. (2013) argued that maintaining the sustainability of rice farming in the face of water scarcity^6^ coupled with stagnant or declining yield was challenging. In South and Southeast Asia, rice is widely established by transplanting. The old method, known as the puddled transplanted rice (PTR) establishment method, involves growing seedlings in a nursery bed and later transplanting them into the main field. The second method of growing rice is the direct-seeded rice (DSR) method. This low-cost establishment technology provides an opportunity to improve water and environmental sustainability (Khush, 1995; Joshi et al., 2013).

Sustainable production methods such as direct-seeded rice can increase production even as land availability decreases and the frequency of water shortages increases. The direct-seeded rice establishment method involves broadcasting, drilling, or dribbling seeds into dry or moist soil (Chauhan et al., 2015). Mishra et al. (2017) analyzed the impact of adopting direct-seeded rice on rice yield and production costs. The authors analyzed a sample of 537 rice farmers in Punjab and Uttar Pradesh, India. They used endogenous switching regression methods to account for biases from observable and unobservable factors. The authors found that smallholder rice farmers in the two states who adopted the direct-seeded rice method of seeding increased rice yield by 7% and decreased rice production costs by about 8%. Direct-seeded rice farmers significantly reduced their irrigation costs (by INR 287/acre or USD 5.37/acre) and their land preparation costs (by INR 958/acre or USD 17.94/acre^7^) compared with their counterparts.

China is the largest producer and consumer of rice. In recent years, China has faced labor shortages, soil erosion, decreasing soil fertility, higher variability in weather, and increased demand from consumers for high-quality rice. The scarcity of water threatens China's rice production. These problems have led to a surge in sustainable rice production techniques in academic and public discourse, including options to grow rice with less labor and lower water intake.^8^ Thus, Sha et al. (2019), using plot-level data from four southern provinces in China and the endogenous switching regression method, investigated the impact of adopting direct-seeded rice on rice yield and net rice income. The authors found that if farmers growing puddled transplanted rice had adopted the direct-seeded rice method, they could have increased rice yield by 3.1% and their net revenues by 66%. Indeed, these two studies, conducted in China and India with the largest populations and the most rice farmers, revealed that the direct-seeded rice establishment method could be a boon for rice farmers' economic well-being in rice-producing nations of South and Southeast Asia.

### 4.2 Other agronomic practices

Local production is insufficient to meet the demand of the increasing population in Africa. However, inland valleys, which are numerous in West Africa, have biophysical conditions suitable for intensifying and expanding rice production (AfricaRice, 2010). Barriers to increasing production in inland valleys include poor water control, weed invasion, low soil fertility, increasing soil degradation, and exposure to the risk of water-borne diseases, among others. Initiated in Benin and Togo by AfricaRice in 2010, the smart-valley approach was introduced through the Sawah, Market Access, and Rice Technologies for Inland Valleys (SMART-IV) project to help overcome soil fertility problems and improve water management to enhance rice production. The smart-valley approach applies land leveling, bunding, and puddling in combination with good water management (AfricaRice, 2015). In a recent study, Arouna and Akpa (2019) estimated the effects of using the smart-valley approach^9^ on farming in Benin and Togo. The authors analyzed data from 590 rice-farming households there. They used the local average treatment effect approach to control for biases from observable and unobservable attributes (Abadie, 2003). They found that farmers who adopted the smart-valley approach increased their rice yield by about 0.9 tons per hectare. The income of rice farmers who adopted this approach increased by USD 267 per hectare. The study argued that adopting smart-valley technology also would help farmers adapt to climate change.

### 4.3 Post-harvest and other technologies

Post-harvest technologies such as the ASI rice thresher-cleaner have been an essential advance for rice farmers. The ASI thresher is a motorized system used to thresh rice in any terrain, including dry, waterlogged, or rough surfaces. It can be transported on vans or attached as a trailer. This technology was developed by collaborative and adaptive research involving two CGIAR centers (AfricaRice and the International Rice Research Institute); national partners such as the Senegalese Institute for Agricultural Research and the Senegal River Valley National Development Agency; artisans; agricultural machinery factories; farmers; and farmers' organizations. Thus, the ASI's initials are an acronym of the three organizations that developed this post-harvest technology. The ASI thresher is an improved post-harvest technology developed and released in 1997 that is important for reducing post-harvest labor bottlenecks and improving the quality of rice for irrigated rice farmers in the Senegal River Valley. In a recent study, Ogwuike et al. (2021) examined the association between the adoption of ASI thresher technology and credit use in Senegal. The authors used a sample of 194 rice farmers and the propensity score matching method^10^ to account for biases from observable attributes. They found that the adoption of ASI technology increased the total amount of money that rice-farming households borrowed (from 194,000 to 432,000 West African CFA Franc or from USD 347.05 to USD 772.81). The authors argued that adopting technologies helps build farmers' credit portfolios. They also recommended implementing strategies that involve increasing the adoption of the ASI thresher.

### 4.4 Information and communication technology and decision making

Today's fast-paced life and information, enabled by booming mobile, smartphone, wireless, and internet information communication technology (ICT) and the Internet of Things (IoT), have found a base even in poor smallholder farms and farming activities (World Bank, 2021). It should be noted that ICT-enabled services often use multiple technologies to provide information. In resource-constrained environments such as developing and emerging economies, ICT providers use satellites or remote sensors to gather data on temperatures and use internet services to store and process large amounts of data. This model is being used to provide rural farmers with localized forecasts for weather-related events, prices, farming practices, and nutrient management. Information relayed through this ICT prevents crop losses by protecting smallholders from droughts, floods, and hurricanes. Farmers also can obtain information on market conditions, location of local markets and prevailing prices to maximize their income (FAO, 2004).

To this end, the International Rice Research Institute developed the web- and mobile-based digital support tool Rice Crop Manager (RCM; Buresh et al., 2019), and AfricaRice developed the Android-based application RiceAdvice (Saito et al., 2015) to provide farmers with personalized nutrient management recommendations (type, quantity, and timing of fertilizer). The Rice Crop Manager has been adopted in the Philippines, India, Indonesia, Myanmar, and Bangladesh. Researcher-managed and on-farm evaluation trials in the Philippines (Buresh et al., 2019; Banayo et al., 2018) and India (Sharma et al., 2019) demonstrated that farmers achieve significantly higher yield and income by adopting Rice Crop Manager recommendations. However, scaling Rice Crop Manager is challenging because of several concerns related to the “enabling environment, human resources and capacity, partnership, technology platform, and monitoring, evaluation, and learning” (Florey et al., 2020).

RiceAdvice, on the other hand, examines data about farmers’ local context to advise them on fertilizer application and agro-management practices (Saito et al., 2015). In a recent study, Arouna et al. (2021) estimated the effects of using a mobile application that provides personalized advice on rice nutrient management. About 700 Nigerian rice farmers participated in a randomized control trial experiment (Arouna et al., 2021). The authors calculated the adoption impacts using OLS and covariance (ANCOVA) analysis. They found that farmers using the app increased rice yield by 7% and profit by 10% without affecting total fertilizer use. Using ICT such as mobile technology allows policymakers to move away from one-size-fits-all advice toward personally tailored farming practices and agronomic choices.

### 4.5 Training and institutions

Agricultural training is another potential method to diffuse new technology (Anderson and Feder, 2007; Feder et al., 1985; Otsuka and Larson, 2015). However, training all farmers would be quite expensive for companies and the government. Agricultural extension services in West Africa face significant challenges. Smallholders cannot access timely information on new technology and cannot achieve food security (Zossou et al., 2020). Social networks have become a more popular way of collecting information on new technologies (Conley and Udry, 2010; Shiferaw et al., 2015; Ward and Pede, 2014). This is especially true for adopting stress-tolerant crops because the benefits become visible only under flooding and drought stresses (Yamano et al., 2018).

Social learning or “learning from others” is another form of extension outreach that could be used to diffuse technology. ICT could be used for social learning in the farming sector. The ICT decision-making model that has been tested in Africa is the farmer-to-farmer extension program. This program has two stages. In the first stage, in 2009, the program trained a group of farmers (called key farmers) by instructing them about rice cultivation technologies provided by the Japan International Cooperation Agency and the Ministry of Agriculture Training Institute of Tanzania. In the second stage, the key farmers used the knowledge acquired in the first stage to train other farmers (labeled ordinary farmers). The program implemented three rounds of surveys of rice farmers in Tanzania in 2010, 2011, and 2012. Nakano et al. (2018) estimated the effects of the farmer-to-farmer extension program using difference-in-difference (DiD) models and accounting for spatial dependence with spatial econometrics techniques. The authors found that the farmer-to-farmer extension training program increased the yield of critical farmers (defined as farmers who received intensive pre-season training at a local training center) by 3.1–5.3 tons per hectare and the yield of ordinary farmers by 2.6–3.7 tons per hectare (Nakano et al., 2018). The study argued that farmer-to-farmer extension programs are cost-effective alternatives to conventional ones.

Smallholders often are located in rural areas lacking information on markets and marketing conditions. Farmers usually sell their products to local traders, who often have access to market prices and information. Imperfect information leads to exploitation in terms of profitability and time management. To help farmers in sub-Saharan Africa, AfricaRice and the National Agricultural Research Institute of Benin developed a program to link producers more efficiently with traders. This program consists of training producer groups in financial management, conflict management, and group marketing. The training enabled the formation of collective marketing groups (AfricaRice, 2015). A study suggests that participating in collaborative marketing improves product quality and access to credit (Hellin et al., 2007). However, establishing and managing cooperative marketing organizations presents challenges such as defining rules of operation and monitoring compliance with regulations (Hellin et al., 2007). Using data on rice farmers from Benin, Arouna (2018) estimated the effects of participating in collective marketing platforms on household income and food security. Arouna (2018) used a local average response function approach to minimize biases. The study found that participation in collective marketing increased the income of rice farmers by USD 148 per hectare on average.

In recent years, contract farming has emerged as a popular vertical coordination mechanism to encourage this coordination (Otsuka et al., 2016). Production contracts can shift farming risk and provide for smallholder agricultural households' transportation, storage, and capital needs. Smallholders producing high-value and low-value crops in many Asian countries have used contract farming arrangements. Empirical evidence (Meemken and Bellemare, 2020; Mishra et al., 2018) from Africa and Asia shows that contract farming positively impacts growers’ profit, yield, and technical efficiency. A recent study by Arouna et al., 2021a, Arouna et al., 2021b investigated contract farming by rice-processing firms in Benin. The authors implemented a randomized control trial using 953 farmers. The program offered rice production contracts to smallholder farmers by working with a rice-processing firm. Farmers in the treatment group received (a) the control contract, (b) the control contract combined with extension services and training, or (c) the control contract, extension training, and input loans. The authors used OLS and ANCOVA models to measure the impacts of the contracts on rice yield. Arouna et al., 2021a, Arouna et al., 2021b found that participation in contract farming increased the area planted with rice by 23% and increased yield by 29%. The authors also found that contracts increased income by 50%. Arouna et al., 2021a, Arouna et al., 2021b noted that “the simplest contracts have impacted almost as large as contracts with additional attributes.” Additionally, the adoption and promotion of contract farming in rice-producing nations could be used to encourage specific nutrients and best management practices (Flor et al., 2021). Indeed, contract farming is a form of socio-technical system with rules and rice traders, input suppliers, and other stakeholders implementing the regulations and arrangements.

In summary, studies show that: (1) adoption of natural resource management technologies, like direct seeded-rice and the smart-valley approach, increased rice yields and income of rice farmers and reduced water usage; (2) adoption of rice thresher technologies, information management technologies, including mobile applications, increased borrowing capacity of farmers and yields and incomes of rice farmers and (3) social learning and contract farming have increased rice yields and income of farmers in African countries.

## 5 Limitations and knowledge gaps

The current study has several limitations. First, it does not cover the whole range of rice research and development and technologies of the CGIAR rice research. Therefore, the reviewed impacts cannot represent the whole range of activities and investments made in these programs. Second, most of the studies used cross-sectional data to assess the impact of innovations on the yields and incomes of smallholders. Recent studies fail to show the extent (years after adoption) of the technologies' positive effects on households in improving their livelihood. Also, the innovations’ impact on other outcomes such as resource use efficiency, social and environmental impacts, risk reduction, management abilities, and potential tradeoffs remains limited. Third, the methodologies used in the assessment studies also are limited. Only one study used the randomized control trials method, the gold standard in ex-post impact evaluations. New stress-tolerant varieties recently released in Asia and Africa have not been evaluated through randomized control trial experiments. Although such experiments often require significant investments and resources, implementations are essential before developing large-scale dissemination strategies. Fourth, some studies have not reported baseline data to identify changes over time with and without new rice technology. With the resource constraints, recurrent surveys could not be established to monitor changes over time. As a result, panel surveys for impact assessment were limited among the studies reviewed. Fifth, this article did not include studies focusing on the effects of technologies in other impact domains such as nutrition, health, climate change adaptation, gender equality, and environment. Sixth, rice varieties traditionally relied mainly on the morphological descriptors and naming of breeders and farmers. However, environmental conditions affect morphological descriptors, and naming is error-prone, particularly in informal seed systems in many rice-growing areas.

Thus, future studies should involve dynamic or panel datasets to conduct ex-post assessments of rice technologies. For instance, although salinity-tolerant varieties have been broadly adopted in Vietnam (Paik et al., 2020), their yield advantage has not been revealed yet because of the absence of severe salinity intrusion in the years subject to the study. Including years with severe salinity problems in the analysis may reveal the advantage of this innovation. Second, future studies on impact assessment should use the randomized control trial method to precisely measure the impact of rice technologies developed by the CGIAR system. Also, randomized control trials performed on different modes of dissemination will allow researchers to identify appropriate strategies for the large-scale adoption of varieties. Third, over the past two years, the COVID-19 pandemic has affected several outcomes in the rice sector, and farmers have adopted several adaptation strategies to cope with this pandemic. Future studies could examine the impacts of COVID-19 on technology adoption, yield, rice income, gender, and farmers’ livelihood. Fourth, survey data and future studies should monitor the adoption of new rice varieties and combine it with DNA fingerprinting of collected rice seed samples to cross-validate survey results. Finally, from a methodological standpoint, as a general trend in the impact assessment literature, most studies tend to show that innovation affects households, communities, and regions the same way, which is not always the case. This often is reflected through fixed coefficients in regression models. Despite the progress in Geographical Information Systems, remote sensing, and spatial econometric techniques, spatial heterogeneity rarely is considered in models for impact assessment. New impact assessments of innovations should consider accounting for spatial dimensions in their methodological approach to examining impacts.

Climate-smart technologies and digital tools for agricultural and institutional innovations (such as business models for youth entrepreneurship) are emerging technologies on which ex-post impact analysis needed to be focused. Regarding the new studies, a future ex-post impact assessment should concentrate more on assessing the impact at large and the impact long term to contribute to the evaluation of sustainable development goals such as environmental health, biodiversity, and mitigation of climate change.

## 6 Conclusions

The review of selected ex-post impact assessment of the adoption of rice-based innovative technologies performed in this study showed that improved varieties, agronomic practices, post-harvest technologies, information communication technologies and decision tools, training and institutions had a significant positive impact among smallholder rice producers in Asia, Africa, and Latin American countries. This review provides evidence of the benefits that smallholders accrued from the adoption of the promoted innovations. Although studies here show rice innovation's positive effects on income and yields, the impacts of several technologies are still largely unexplored. These include, but are not limited to, newly released stress-tolerant rice varieties, hybrid rice, improvements in water management, pest management, mechanization, and seed systems. Moreover, several rice regions in Asia, Africa, and Latin America deserve further attention from ex-post impact assessment. The lack of longitudinal data, particularly the absence of baseline information and adoption figures for several innovations, has had major implications on the research methods and has caused limitations in area coverage for most impact assessment studies.

Most of the technologies discussed in this review were designed for small, resource-poor farmers with low productivity and low potential. Indeed, the rice technologies discussed above are for resource-poor (small scale) farmers, but we should not underestimate the importance of small-scale farmers in feeding the world. For instance, 60% of rice consumed in Africa is produced by small-scale farmers, and 40% of importation in Africa is produced by small-scale farmers in Asia. Small-scale farmers also are leading production in many Asian countries such as India, Thailand, and the Philippines. Rice innovations have increased the food security of low-income families, increased incomes, and enhanced livelihood opportunities for millions of smallholders in major rice-producing countries in Asia and Africa and in some Latin American countries. Thus, we cannot diminish the role of smallholder producers and their access to technologies may be restrained if initiatives like the CGIAR do not occur at the right time. Additionally, smallholders still matter because they are more efficient in production, given the returns to scale and the inverse relationship between farm size and productivity. Smallholders also are considered stewards of natural resources and the environment.

The rice innovations achieved by CGIAR systems will not directly help feed the world but would indirectly help by providing a livelihood for farming households. Facilitating the enabling environment (for technology adoption) seems critical to smallholders and to the success of CGIAR's rice research program. Moreover, increasing population and reduced landholdings in many rice-consuming countries will require increased production from existing large farmers. Rice innovations also could be suited for large farms in the United States, Japan, or southern Brazil. Rice innovations that are fruitful to large rice farmers eventually will help feed the world. Future ex-post impact assessment studies could focus on rice technologies' large-scale impact on rice production in feeding the world.

## Declaration of competing interest

The authors declare that they have no known competing financial interests or personal relationships that could have appeared to influence the work reported in this paper.

## Footnotes

1 Formerly Consultative Group for International Agricultural Research.

2 Impact studies on agronomic field trials are not included.

3 The score was calculated for four different seasonal periods to capture variations in food availability. The score is the sum of weighted food group times the number of days each food group is consumed during a period of one week.

4 USD 1 = PHP 40.71.

5 CURE is a network of 10 Asian countries to support farmers living in unfavorable rice-growing environments: Seven countries are in Southeast Asia (Cambodia, Indonesia, Laos, Myanmar, the Philippines, Thailand, and Vietnam), and three are in South Asia (Bangladesh, Nepal, and India).

6 The International Water Management Institute estimated that, by 2020, one-third of the Asian population would face water shortages.

7 In 2012, USD 1 = INR 53.40.

8 Agronomic studies claim that the DSR method enhances land productivity and labor efficiency (Kumar and Ladha, 2011).

9 The authors argue that, because of the low costs of dissemination and participation related to the smart-valley approach, it was well received. The total area with the smart-valley approach increased from 110 ha in 2012 to 474 ha in 2014.

10 The authors used four propensity score matching techniques (nearest neighbor, caliper, stratification, and kernel).
